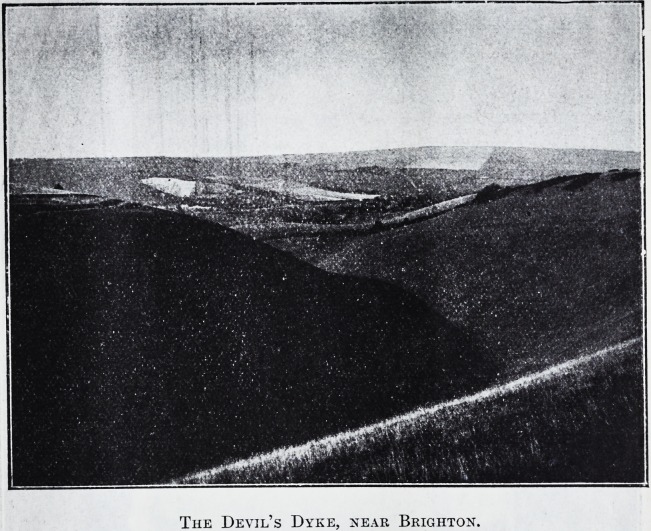# The Public Health: Interviews with Local Authorities: Brighton

**Published:** 1924-02

**Authors:** 


### THE PUBLIC HEALTH.

### INTERVIEWS WITH LOCAL AUTHORITIES.

### XV.—BRIGHTON.

THE name of Brighton is in itself a considerable asset to the town. Whatever its exact origin —whether or no its comparatively recent name of Brighthelmstone is rightly attributed to a Saxon Bishop named Brighthelm, who seems to have accompanied an army in the neighbourhood in A.D. 447 with other than peaceful intentions—the present name undoubtedly suggests sunshine and health and good cheer. Brighton, in fact, has a reputation for these things to which her people and her Council have to live up; they accept this and tell you that "Brighton for health and pleasure all the year round" is the keynote of the town.

Councillor W. E. Parry, Chairman of the Brighton Public Health Committee.

Dr. Duncan Forbes. M.B.E., Medical Officer of Brighton.

#### The Round of Pleasures.

As to her pleasures, we cannot do more here than glance at her unceasing round of attractions which are designed for her multitudes of visitors, as well as for the 135,000 people who make their home here. The theatres, the cinemas, the Aquarium, the fine shops, the first-rate concerts on the pier and on the front, and in the Pavilion grounds and the Dome; the motor chars-à-banc, the boats and the little tramway that runs through the sea—these are all contributory to that cheer and relaxation which are the handmaidens of health, with which we are more immediately concerned. That remarkable building, the Royal Pavilion, erected by King George IV. when he was Prince of Wales, which seemed such an appropriate setting for the wounded Indian soldiers during the War, has its own tale of pleasure of bygone days. But you must go elsewhere than to the official guide for the story. "We do not propose to detail the history of the Royal Pavilion, or to comment on the conduct of its first Royal owner and the company he kept there," writes the discreet Director of Publicity. Ah, well!

#### The Council's Responsibilities.

Those who revel in the breezes that blow over the glorious Downs which lie behind the town, or play golf on the splendid municipal links, or bask in the sunshine of the sheltered Madeira Drive, which is so attractive a feature of the Brighton sea-front, and then turn to the various social entertainments for their further delectation, would not readily recognise that for the maintenance of her proud position as "Queen of Watering Places," constant activity and vigilance on the part of the Corporation are needed. A special measure of responsibility in a health resort surely rests upon the Health Committee and their medical officer. The present chairman of that committee, Councillor Parry, and the Medical Officer of Health, Dr. Duncan Forbes, very cordially responded to our invitation to furnish a short account of some aspects of their work.

#### Climate and Infant Mortality.

Brighton, it is claimed, is 10 per cent. cooler in summer than London, and 7 per cent. warmer in winter. The average annual rainfall is 28.5 inches, and it is noteworthy that after the heaviest storm the storm water is rapidly drained off the pavements and the roads dry up. The water supply is excellent, being derived from deep wells in the chalk, and it is under Corporation control. The sewage is run into the sea five miles away to the east of the town. The death rate of 12.57 in 1921 and 14 in 1922, although comparing not unfavourably with the average for county boroughs, really suffers by reason of the fact that aged persons and invalids who settle in the town ultimately load the death rate. The enteric fever incidence in Brighton is only about one-third of that of the country generally. The infantile mortality rate of 79 in 1921 was already much below the average 92 for county boroughs for that year, and dropped in 1922 to 67. They have in Brighton a district midwifery service organised by the Women's Lying-in Hospital from which nearly half the births in Brighton are attended. Special attention is given to pre-natal work with excellent results. There are five infant welfare centres in the town to which the four health visitors send mothers with normal as well as wasting children, so that they may have advice as to diet from the maternity and child welfare doctor. A ward is reserved for wasting infants at the Sanatorium, chiefly for children who cannot get proper attention at home, although, given an intelligent willing mother who will allow herself to be advised, an infant will nearly always do best at home.

Madeira Drive: The Covered Walk.

### The School Clinic—Treatment of Ear Discharge.

The Medical Officer of Health is School Medical Officer with resulting close co-ordination of infant health, welfare, and school work. For instance, any defect in children discharged from the Infectious Disease Hospital is reported either to the school doctor or the maternity and child welfare doctor. All information regarding pre-school life is sent to the school clinic. Operations for tonsils and adenoids are done at the Infectious Disease Hospital in the discharge block, and spectacles prescribed on the school clinic premises, where three school doctors and two school dentists are at work. Dr. Forbes pointed out that they have had great success in stopping ear discharge by zinc ionisation, over 70 per cent. of cures resulting from the treatment. Many authorities are following the lead of London and Brighton in this matter. X-ray treatment for ringworm of the scalp was given with most satisfactory and permanent results.

### The Tuberculosis Scheme.

Having confined himself to tuberculosis work and practically taking no part in other fields of health work in the borough, the Clinical Tuberculosis Officer keeps himself abreast of all the latest advances in treatment, and has the confidence of the profession in his capacity as consultant. This gives every encouragement to the general practitioner to send early doubtful cases for diagnosis to the Tuberculosis Dispensary. A number of cases of tuberculosis of the joint are treated at the Sanatorium with marked benefit and usually with arrest of disease.

### A Club for Phthisis Patients.

Mr. Parry said that one of the outstanding features in their tuberculosis scheme in which Brighton has got ahead of other authorities is the establishment of a club for phthisis patients. Instead of sitting at home the patients can call at the club and rest in the fresh air, see the daily papers, and play billiards and other games. The garden work and the workshop at the Sanatorium are also notable features of the tuberculosis scheme. The recommendation of the medical officer that a municipal workshop should be provided where consumptives could work under ideal conditions, for longer or shorter hours, according to their fitness, is one that would benefit enormously the patients and the public generally, and is receiving the consideration of Mr. Parry and his committee. Dr. Forbes encourages the general practitioner to consult him in doubtful cases of infectious disease, going out personally to see the cases and leaving very little of this work to any assistant. He is also willing to take swabs for them, to take cerebro-spinal fluids at the homes or to take bloods for Widal or Wassermann reactions. This is really a very important feature of the work of the medical officer which in other towns is not always given the attention which it deserves. The number of examinations of all kinds made at the laboratory in 1922 was 3,475. Free immunisation against diphtheria is offered. In the course of the interview Dr. Forbes expressed the interesting view that there is elsewhere much unnecessary disinfection of houses and rooms; he said that in Brighton they are saving money in this matter, and that some other authorities are inclined to follow their lead.

The Devil's Dyke, near Brighton.

### Housing and Sanitary Department.

We can refer only briefly to the work under this head. It is all important, but corresponds in its main features with the work in other towns. Over 800 houses have been built by the Council since the War; the work of slum clearance is constantly exercising the minds of the authorities, who are sadly hampered, said Mr. Parry, by the difficulty of providing the homes which are so essential before the slum dwellers can be disturbed. They have their slums in Brighton as elsewhere, where nothing short of wholesale clearance and rehousing on the site will counteract the unhealthy conditions caused by congestion of buildings, narrowness of streets, lack of air space and ventilation, and the ruinous, damp and derelict condition of the houses themselves. The unexciting but important work of dust clearance and scavenging receives special care in Brighton; defective dustbins are promptly replaced, and in the poor quarters in the summer there is a biweekly, instead of the usual weekly, removal of refuse. The Corporation have had since 1894 their own abattoir; whereas there were then 40 private slaughter-houses there are now 13 only. It is the policy of the Health Committee, said the chairman, to buy out the private slaughter-houses as opportunity offers and ultimately to have all the killing done at the abattoir by the most humane methods.

### "Dr. Brighton."

It is many years since Thackeray gave the town the name of Dr. Brighton, and it has stuck. By stage coach, by Pullman or ordinary train, by motor-car, by motor-omnibus, by bicycle, nay, on foot, the throngs have come, and continue to come, to this medical celebrity in search of health and recreation. The place has been much favoured by Royal visitors, and it is its proud boast that the late King Edward came three times in one year to Brighton for recuperation and reinvigoration. The maintenance of the reputation of so eminent a town rests in no small degree with the Health Committee and their medical adviser, and we came away feeling that it is secure in the hands of the present chairman, Mr. Parry, and of Dr. Duncan Forbes and his staff.

---

### BIRTH- AND DEATH-RATE CHANGES.

Although from the point of view of lower death rates, we can look upon the past year as a healthy one, yet it must be borne in mind that the birth-rate also, 19.8 per 1,000 in the third quarter, was the lowest recorded for any corresponding period, except during the war. Fortunately we can set against this the fact that the infant mortality rate stood at 57—that is 27 per 1,000 lower than the average for the same season over the last ten years. There seems little doubt that this low rate was due to the absence in 1923, and 1922 also, of any really serious epidemic. Therefore, it may not be justifiable to expect such good figures to be permanently maintained, and though much of the improvement is undoubtedly due to better health organisation, good fortune has also come into play.

## Figures and Tables

**Figure f1:**
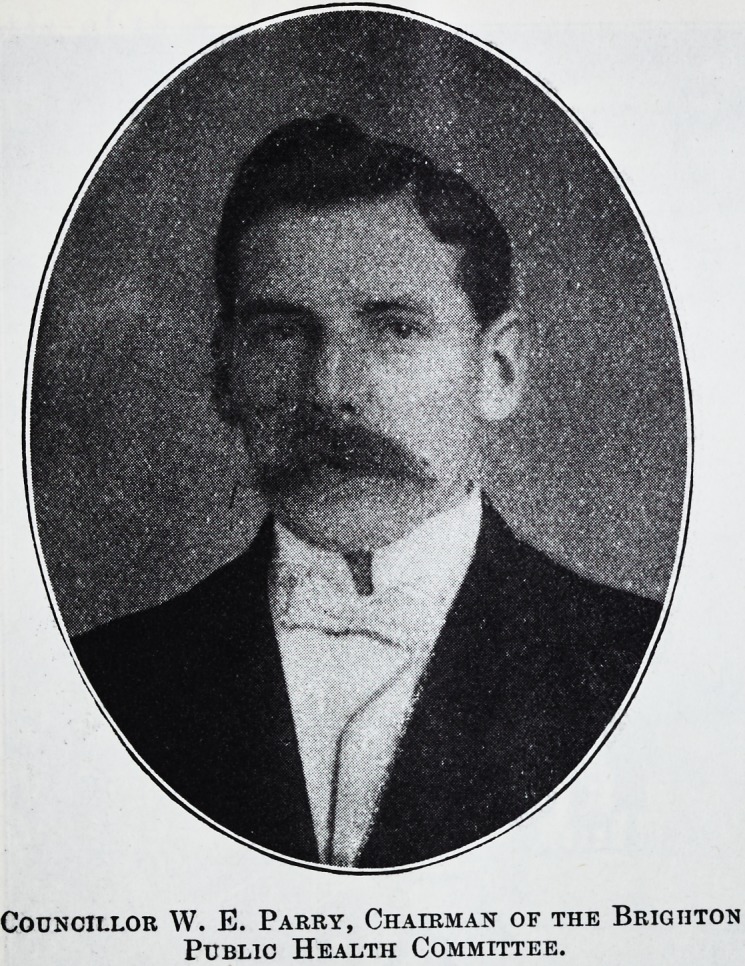


**Figure f2:**
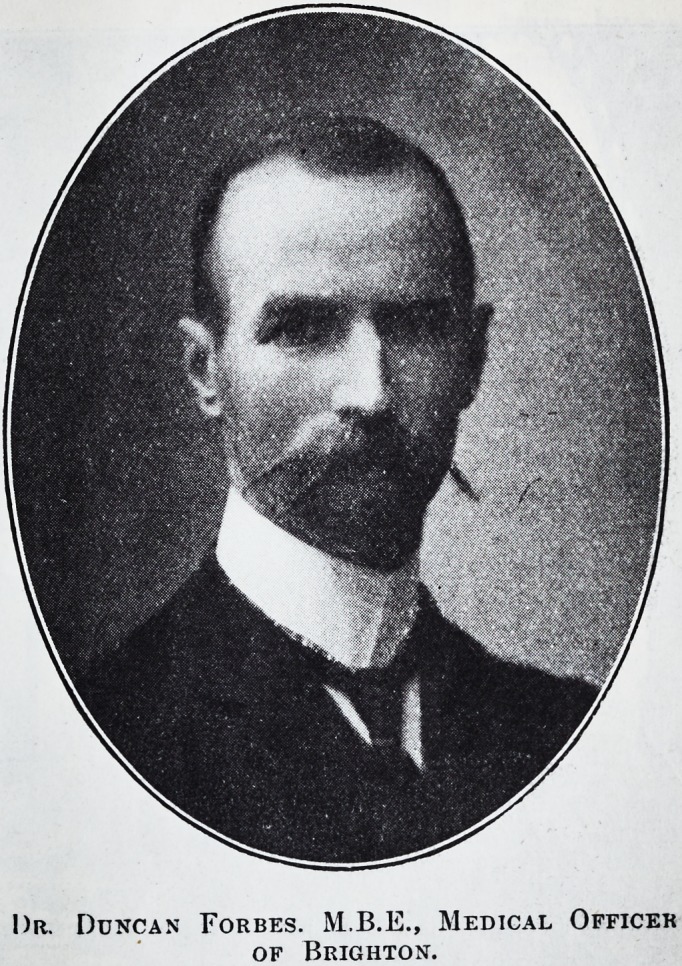


**Figure f3:**
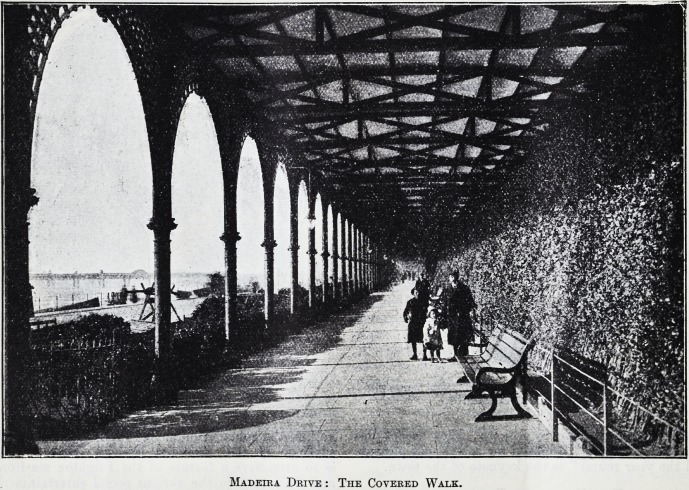


**Figure f4:**